# Interaction between anthropogenic stressors affects antipredator defense in an intertidal crustacean

**DOI:** 10.1093/beheco/arae085

**Published:** 2024-10-10

**Authors:** Laura Coles, Tom Tregenza, Martin Stevens

**Affiliations:** Centre for Ecology and Conservation, University of Exeter, Penryn Campus, Treliever Road, Penryn, Cornwall, TR10 9FE, United Kingdom; Centre for Ecology and Conservation, University of Exeter, Penryn Campus, Treliever Road, Penryn, Cornwall, TR10 9FE, United Kingdom; Centre for Ecology and Conservation, University of Exeter, Penryn Campus, Treliever Road, Penryn, Cornwall, TR10 9FE, United Kingdom

**Keywords:** antipredator, camouflage, noise pollution, shore crab, stressor interaction, temperature

## Abstract

The marine environment is increasingly subject to changes driven by anthropogenic stressors which may alter species’ key behaviors and impact phenotypic plasticity. Such stressors rarely occur in isolation, yet our understanding of how simultaneous stresses affect marine organisms is limited. Here, we study the combined impacts of a major global stressor, temperature increase, and a local stressor, anthropogenic noise, upon key defensive traits of the shore crab, *Carcinus maenas*. We tested the color change and behavioral responses of crabs in relatively colder and warmer water, and in the presence of natural ambient or ship noise. Using image analysis and a model of predator vision, we demonstrate that crabs change color, and improve camouflage, fastest in warmer water in the absence of anthropogenic noise. When anthropogenic noise was present, it adversely impacted crab color change and camouflage, to the extent that the accelerated change due to temperature was negated. In addition, anthropogenic noise affected *C. maenas’* behavior, reducing the likelihood and increasing the latency of antipredator response to stimuli. This reveals an interaction between the 2 stressors, with the combination of temperature and noise eliciting different biological responses compared with the effects of each stressor in isolation. Our study demonstrates how such interactions between anthropogenic stressors may impact marine life.

## Teaser

Outcomes of stressor interactions may be complex and can have dramatic consequences on behavior of individuals. Here, we show that the interaction between raised temperatures and anthropogenic noise pollution affects the antipredator defenses of the European shore crab. In a laboratory setting, we reveal an interaction between the two stressors in their impact on crab camouflage, as well as an impairment in predator avoidance behaviors under conditions of anthropogenic noise pollution.

## Introduction

Marine species are increasingly subject to complex environmental changes wrought by exposure to multiple stressors, often anthropogenic in source (Halpern et al. 2008). The effects of such stressors upon organisms may be impacted by biological and environmental factors, such as the trophic level of species, their life-history stage, morphology, distributional range, and habitat heterogeneity, making them difficult to predict (Parmesan 2007; Sunday et al. 2015). Due to the ever-growing range of anthropogenic sources of stress on the natural environment, and the difficult task of accurately monitoring marine species’ responses, particularly in situ, large gaps remain in our knowledge of the impacts of multiple stressors. This could give rise to serious cause for conservation concern, especially where interactions between stressors cause unpredictable consequences and so-called “ecological surprises” (Paine et al. 1998).

Generally, stressor interactions may be classified into one of 3 groups: synergistic, additive, or antagonistic. The combined effect of 2 stressors on an organism may be equal to (additive), less than (antagonistic), or several magnitudes greater than (synergistic) the sum of their individual effects (Folt et al. 1999; Crain et al. 2008; Côté et al. 2016). In all scenarios, failure to consider stressor interactions may lead to misguided conclusions and ineffective subsequent management actions. For example, coral health may be affected by turbidity, UV exposure, water temperature, and fishing activity. Corals experiencing increased turbidity may have higher rates of disease (Pollock et al. 2014), yet when facing high UV exposure, increased turbidity protects corals from the harshest UV conditions, reducing rates of bleaching. As such, managing this stressor could lead to overall decline of coral health (Anthony et al. 2007). In terrestrial environments, lack of consideration of stressor interaction may lead to inaccurate estimates of population declines (Didham et al. 2007), poor biodiversity policy planning (Brook et al. 2008), or unreliable predictions (Hanson et al. 2005). This is particularly true with combinations of local stressors (stressors affecting a limited range from their source) and global stressors (stressors with global causes such as climate change), where it is often most feasible and practical to manage local stressors but this may have adverse net impacts if interactions with global stressors are not fully considered. In all cases, stressor interaction directly impacts management outcomes (Brown et al. 2013; Crain et al. 2008).

Sea surface temperature is a global stressor, driven by increasing and unprecedented rates of climate change and leading to alterations in species behavior and physiology (Halpern et al. 2015). Increases in temperature have been linked to higher susceptibility to disease (Shish and Ducklow 1994), shifts in species range (Helmuth et al. 2006), and altered growth and timing of reproductive events, potentially leading to temporal mismatches between predators and prey (Lawrence and Soame 2004). Organisms in intertidal zones may be particularly vulnerable to extreme impacts related to climate change since, although they have evolved to cope with a challenging environment, they may already be close to their physiological limits in dealing with environments that fluctuate greatly in temperature, light, salinity, and community ecology (Hewitt et al. 2016). Impacts of temperature may not always be unequivocally positive or negative. In fact, whether species respond to raised temperatures with stress may vary according to additional factors, including seasonality, community co-tolerance to other stressors, microhabitats (e.g. high versus low shore species in intertidal inhabitants), or acclimation period. Positive effects may occur with increasing temperature until a thermal threshold is reached, after which stress is exhibited (Brown et al. 2013). Due to this, and conforming to the definition of a stressor as a factor altered outside of a natural range by human activity, we refer to rising temperature as a stressor throughout this paper.

Noise pollution represents a pervasive local stressor in both terrestrial and marine environments, with sound from commercial shipping having wide ranging, chronic, impacts upon even species relatively isolated from coastal urbanization (Slabbekoorn et al. 2010). Shipping noise has caused a global rise in ocean sound levels of 1 to 2 dB every year (Ross 2005) and often produces low-frequency sounds that overlap with the auditory detection ranges of a plethora of marine species (Popper et al. 2001; Hughes et al. 2014; Radford et al. 2022). In invertebrates, ship noise has been associated with increased stress, reduced reproductive success, and altered growth and foraging behaviors, color change and camouflage, and mating behavior, resulting in potentially severe impacts (Chan et al. 2010; Wale et al. 2013a, b; Nedelec et al. 2017; Carter et al. 2020; Rising et al. 2022).

One invertebrate that has been increasingly studied with regards to impacts of noise pollution is the shore crab, *Carcinus maenas*. This intertidal species is native to European coasts, and highly invasive in many other locations. Juvenile shore crabs are extremely variable in carapace coloration and patterning, and demonstrate the ability, when transplanted onto different substrates, to alter their appearance to achieve a better match to the background (Stevens et al. 2014a, b; Carter et al. 2020). Exposure to anthropogenic noise pollution has been demonstrated to result in both physiological and behavioral alterations in adult *C. maenas.* When exposed to playback of ship noise, shore crabs increased their oxygen consumption, indicative of a higher metabolic rate and increased cardiovascular activity, and suggestive of stress (Wale et al. 2013). In addition, adult shore crabs display reduced foraging rates, impaired response to a simulated predatory attack, and take longer to retreat to shelter under ship noise than ambient noise conditions (Wale et al. 2013). Juvenile *C. maenas* have also been shown to respond slower to predatory threat under conditions of noise pollution (Hewitt et al. 2016). Furthermore, crabs exposed to ship noise had significantly reduced levels of color change, and poorer camouflage after several weeks compared even to crabs that changed color under ambient noise played at the same intensity (Carter et al. 2020). In the same study, crabs also molted less often and had reduced size changes per molt under ship noise than ambient noise (Carter et al. 2020). Furthermore, recent work has shown that male shore crabs demonstrate reduced mating behavior toward dummy crabs soaked with female pheromones when in the presence of ship noise versus ambient noise (Rising et al. 2022).

*C. maenas*’ success as an invasive species has previously been linked in part to its wide thermal tolerance range (Kelley et al. 2015), and previous studies have aimed to quantify impacts of temperature changes on shore crabs. Larval development occurs between 10 °C and 23 °C, with an adult thermal tolerance range encompassing 0 °C to 30 °C across the species’ full geographic range, with different populations acclimated to higher or lower temperatures (Compton et al. 2010; Kelley et al. 2013, 2015). At temperatures higher than its typical local range, *C. maenas* exhibits a stress response, with altered oxidative defense, cellular respiration, and phosphorylation (Rodrigues et al. 2015). This may depend on specific environments inhabited by populations of crabs, with 25 °C close to the thermal limit of crabs in their native range (Tepolt and Somero 2014; Nancollas and McGaw 2021). Studies into impacts of temperature on camouflage ability have demonstrated that the rate of crab brightness change (and subsequent camouflage ability) increases with increased temperature (Powell 1962; Mynott 2019). However, the efficacy of this may be affected at higher temperatures, for example, at 25 °C, crabs on light substrates did not change brightness to match their background (Mynott 2019), and at 30 °C white pigment dispersed regardless of background color (potentially due to an attempted albedo effect) (Powell 1962). These studies were also relatively short term (a few weeks maximum) and additional thermal stress (or acclimation) may occur over longer periods.

Here, we study the interaction between these 2 commonly occurring stressors, one local and one global: temperature change and noise pollution. We present their combined impact on key defensive behaviors of juvenile shore crabs. Using a factorial experiment in a laboratory setting, we test how appearance change, growth, and behavior is impacted by the individual and combined effects of ship noise and raised temperatures, and whether the stressors act additively, antagonistically, or synergistically. Our study presents the first exploration of the impacts of a combination of local and global stressors upon the camouflage and antipredator behavior of an intertidal crustacean.

## Methods

Juvenile *Carcinus maenas* were collected from mudflats alongside the tidal Penryn River creek, Penryn, UK (50.168944, −5.097639) at low tide, between February and October 2019, and transported back to the University of Exeter’s Penryn Campus, Cornwall, UK, where all experiments were carried out. *Carcinus maenas* is not a protected species in the United Kingdom and all work was carried out in accordance with the University of Exeter’s Ethics policy (application no. ECORN001803). Crabs were held in glass aquarium tanks filled with dechlorinated saltwater mixed to a salinity of between 30 and 35 ppt (Aquarium Systems Instant Ocean Salt, Instant Ocean, Blacksburg, Virginia), with filters and temperature controlled by chilling units. Initial tank temperatures were maintained at 14 ± 1 °C. Tanks were illuminated by 2 aquarium lights (TMC Grobeam, Aquaray)—one UV and one natural white light, on a 12:12 cycle. Individual crabs were housed within holding tanks, in individual black PVC housing units with fine grade 2 mm mesh on the base and top, allowing water flow and noise transmission. Crabs were initially kept on black aquarium gravel to mimic the dark substrates of the collection location.

Control treatments were based on previous similar experiments (Mynott 2019; Carter et al. 2020), with 14 ± 1 °C considered the control temperature, and 24 ± 1 °C the raised, experimental treatment, while Ambient noise playback was considered the control treatment, and Ship noise playback the experimental, giving rise to 4 conditions: (1) “Cold-Ship,” (2) “Hot-Ship,” (3) “Cold-Ambient,” and (4) “Hot-Ambient.” Total sample sizes for each treatment were the following: Hot-Ship: *n* = 50, Cold-Ship: *n* = 64, Hot-Ambient: *n* = 52, and Cold-Ambient: *n* = 56. Both experiments used the same group of crabs and thus the same sample sizes. All crabs were held for 1 wk to acclimatize, and while the temperature in the “hot” treatments was raised, prior to the start of the experiment. A low temperature of 14 °C was chosen to resemble a yearly average temperature at the collection region, and a high temperature that was at the upper end of temperatures experienced by crabs naturally.

For the sound treatments, recordings taken at UK ports were provided by (Wale et al. 2013a, b) of ambient background noise and the sounds of a ship passing at an approximate distance of 200 m (SOM). The 6 recorded tracks were individually modified in Audacity(R) to play at a comparable amplitude. Throughout the ambient treatments, ambient soundtracks played continuously, in an unpredictable order, while ship noise treatments consisted of this same ambient noise playback, with the addition of a ship noise track played once every hour, again in an irregular order. Sound playback occurred through a UW30 underwater speaker (University Sound Diatran Omni-directional Underwater Loudspeaker, 100 to 10,000 Hz), suspended above the center of the tank, using MP3 players (RUIZU X02 MP3 Player, 8GB) connected via an amplifier (Kemo Electronic; 18W; frequency response: 40 to 20,000Hz). Spectral quality of sound playback was assessed using recordings of in-tank sound levels. Soundtracks were standardized among treatments. Although we do not have calibrated sound measurements for in-tank playback, calibrated information for the original recordings is available (Wale et al. 2013b). Furthermore, we note that previous studies have demonstrated that negative responses to ship noise are exhibited across different amplitudes (Carter et al. 2020), and that actual in situ noise levels will vary in the wild owing to different ship types, passing distances, and additional environmental factors.

### Experiment 1: Camouflage and stressor interaction

#### Photography and Image analysis

To monitor change in crab carapace appearance under the 4 treatments, crabs were photographed for the first time following the 1-wk settling period (“starting appearance”), during which they were held on dark substrates. At the start of the experiment, crabs were placed in individual white PVC housing units (as above) lined with 15 mm depth of white gravel substrate. At this point, sound treatments commenced. For their final appearance, we photographed crabs after 6 wk. Crabs were photographed alongside a 93% and a 7% photographic reflectance standard (Spectralon, Labsphere), and photographs were taken using both a UV band-pass filter (Baader U filter, 300 to 400 nm) and a human visible filter (Baader UV/IR filter, 400 to 700 nm). Photographs of the white substrate were also taken, to be analyzed for background luminance. Image analysis was carried out in Image J (version 1.52k, National Institute of Health, NIH), using the MICA toolbox plug-in (version 1.22, Troscianko and Stevens 2015). UV and human visible images were aligned, linearized, and normalized with regards to the reflectance standards (Stevens et al. 2007; Troscianko and Stevens 2015).

To understand how changes in carapace luminance (perceived lightness) may correspond to real world camouflage against predators, images were analyzed with respect to an avian predator vision model (Peafowl, *Pavo christatus*). *P. christatus* has a violet shifted visual system (Violet Sensitive, VS) capable of perceiving UV light (Hart 2002; Ödeen and Håstad 2003), and provides an ecologically relevant visual system model, comparable to many avian potential predators of juvenile shore crabs (Crothers 1968). Multispectral images were analyzed using the Batch Multispectral Image Analysis function in the MICA toolbox, employing a highly accurate polynomial mapping technique (Stevens et al. 2007; Pike 2011; Troscianko and Stevens 2015) to convert the images to peafowl luminance values based on predicted double cone values. Background matching was quantified as the absolute difference between crab carapace luminance and substrate luminance value (Stevens et al. 2014).

Crab growth was monitored throughout the experiment, with crab weight, carapace width, and molting being recorded. Size and weight data were collected during the photography process every 2 wk, to minimize unnecessary stress.

#### Statistical analyses

Data were analyzed with GLMs (RStudio version 1.2.5042, RStudio, Inc.) using the Gamma family (for luminance, background matching, carapace width, and weight), and the binomial family (for molt data). Data were evaluated for normality using the Shapiro-Wilk test (Shapiro and Wilk, 1965), as well as by visual inspection of plots, and background match data were transformed (square root transformation), in order to meet the assumptions of a Gamma GLM. A maximal model was initially generated, including temperature, noise, and their interaction, as well as week, crab size (carapace width and weight), and molt behavior (molt- yes/no; number of molts). Following this, candidate models were generated and evaluated using AIC to determine minimum adequate model, where lower AIC values corresponded with models with the greatest statistical support (Burnham and Anderson 2004). Model comparison was used to calculate significance of key terms, using χ^2^ analysis of variance.

Post-hoc testing was carried out using the lsmeans package (Lenth 2016) , with *P*-values adjusted according to the Tukey method for comparing a family of 4 estimates.

A Kruskal–Wallis test (Kruskal and Wallis 1952) was used to assess differences in carapace luminance, crab weight, or carapace width, between Hot and Cold treatment groups, at Week 0 of the color change experiment (following the acclimation week, and before noise treatments commenced). This test was chosen to compare the 2 groups as the data were non-parametric.

### Experiment 2: Impact of stressor interaction on antipredator response

#### Retreat from a simulated predatory event

Following the 6-wk color change experiment (described above), all crabs remained in their holding tanks at their acclimated temperatures (14 °C or 24 °C) to be studied in a series of behavioral trials to measure their antipredator responses under the different treatment conditions. On the conclusion of behavioral trials, all crabs were released to their original collection location.

To measure responses to a simulated predatory event under combined noise and temperature treatments, crabs were monitored in a behavioral trial informed by the work of Wale et al. (2013a, b)and Carter et al. (2020). To create a trial arena, holding tanks were divided in 2 portions—a larger holding area (measuring two thirds of total tank volume) and a smaller trial portion (one third), with the division sound-proofed using a fitted polystyrene dividing wall lined with bubble wrap. A gray plastic tray (100 × 300 × 440 mm) was fitted in the trial portion of the tank as a trial arena. Noise treatment playback followed the protocol for experiment 1, and sound recordings (see above) confirmed that trial soundtracks were not detectable within the holding portion of the tank at any point throughout the trials. The trial arena was lined with a fine layer of mixed sand (< 3 mm depth to prevent burial) and 2 rocks were arranged as a shelter (measuring approximately 50 × 80 × 180 mm) in the far-right corner of the tray. All crabs were exposed to one trial under ship noise and one trial under ambient noise (see above), with a 5-min break in between trials. Presentation order and specific track played were alternated between groups of crabs, with approximately half of crabs presented with ship noise first and the other with ambient noise first. Crabs were placed in the center of the experimental arena inside a 60 × 60 × 60 mm white PVC ring, where they were allowed to acclimate for 1 min. After 15 s, the noise treatment was started. After 1 min had elapsed, the holding ring was removed, releasing the crab. Ten seconds after release, a metal dowel rod was plunged into the water three times (once per second, in the center of the arena), to simulate a predatory attack. The time taken for the crab to respond by retreating to the rock shelter was recorded, with retreat having taken place when the crab had successfully hidden at least half of its body (carapace and legs) underneath the shelter (Wale et al. 2013b; Radford et al. 2022).

#### Statistical analyses

Following the method described above, GLMs were used to analyze antipredator data. The initial maximal model for antipredator response initially included temperature, noise, and their interaction, as well as an interaction between these treatments and prior noise exposure, to investigate potential acclimation to ship noise. Candidate models were selected as above, and post hoc testing carried out using the lsmeans package.

## Results

### Experiment 1: Impact of stressor interaction on camouflage and growth

Background matching (the discrepancy between crab and substrate luminance) was significantly improved in all groups at week 6 compared with week 0 (glm(SQRT): χ^2^_1,432_ = 0.096, *P* = <0.001).

**Fig. 1.**
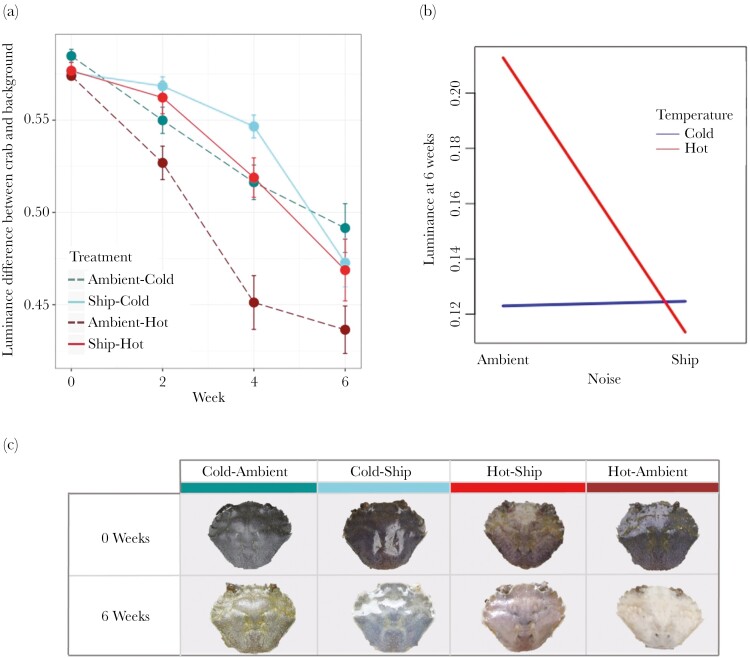
(a) Mean difference in carapace luminance and substrate (background) luminance, with standard error. Lower values indicate a better level of background match. Solid lines denote ship noise treatments and dashed lines denote ambient noise treatments. (b) The stressors anthropogenic noise and raised temperatures interact in their effect on median luminance after 6 wk. (c) Example crab carapace appearance at the start and end of the experimental period by treatment.

Noise and temperature treatments interacted to influence background match of crabs at the end of the experimental period (χ^2^_1,430_ = 0.004, *P* = 0.03), Figure 1b). Crabs held at 24 °C and experiencing ambient noise playback exhibited faster rates of luminance change, and a better subsequent background match than those experiencing ambient noise at 14 °C (*t* = 3.23, *P* = 0.007, Figure 1a,c). However, those crabs experiencing ship noise did not show improved background match when exposed to warm temperatures, with no difference when comparing Hot-Ship and Cold-Ship groups (*t* = − 0.001, *P* = 0.999).

Crabs grew fastest and attained a greater overall size at 6 wk under Hot treatment groups, regardless of noise treatment. As expected, crab molting significantly impacted luminance change (molt y/n χ^2^_1,432_ = 1.07, *P* = 0.052; molt no. χ^2^_1,432_ = 3.29, *P* = <0.001).

There was a small but significant difference in the starting luminance of crabs, which was first recorded following the 1-wk acclimation period, with a higher mean luminance of Hot treatment crabs (Kruskal Wallace; χ^2^_1 (1)_ = 6.28, *P* = 0.01) (see SOM).

### Experiment 2: Impact of stressor interaction on antipredator response

**Fig. 2.**
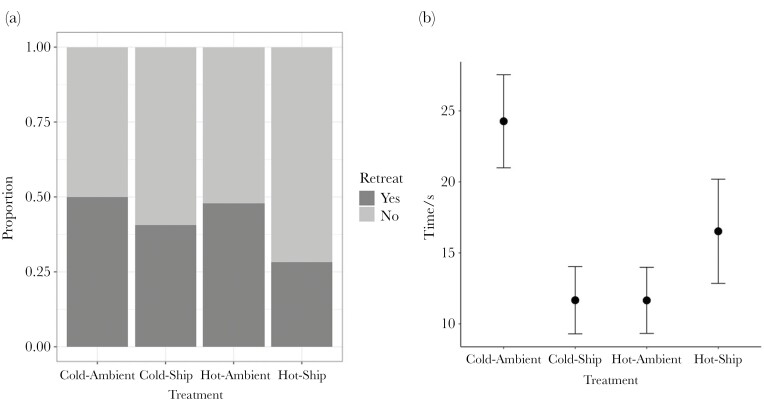
(a) Proportion of crabs exhibiting retreat behavior in response to a simulated predatory attack, or displaying no response. Crabs were generally less likely to retreat under ship noise. (b) Mean time to retreat to shelter among crabs that responded to a simulated predatory attack, with standard error, with crabs under cold water and ambient noise slowest to respond, followed by crabs in warm water with ship noise.

Crabs exposed to ship noise were less likely to respond to a simulated predatory attack by retreating to shelter than those exposed to ambient noise ((glm χ^2^_1,291_ = 4.45, *P* = 0.035), Figure 2a). Of those crabs that responded to simulated predatory attack, time to retreat was significantly impacted by the interaction between temperature and noise treatment ((χ^2^_2,118_ = 10.3, *P* = 0.002), Figure 2b). Crabs exposed to the combination of cold temperatures and ambient noise responded slower than crabs exposed to hot temperatures and ambient noise (*t* = −2.73, *P* = 0.036).

## Discussion

We present the first exploration of the impacts of a combination of local and global stressors upon the camouflage and antipredator behavior of a crustacean. We reveal an interaction between warm temperatures and noise pollution on the camouflage ability of the shore crab *Carcinus maenas* and demonstrate that these combined stressors also affect the speed and likelihood of response to simulated predatory events.

Crabs in all treatment groups improved their background match to experimental substrates, with respect to an avian predatory model. However, the rate and magnitude of this ability was impacted by the thermal and auditory stressors. As expected, those crabs experiencing 24 °C temperatures with ambient noise playback exhibited the highest rates of luminance change and achieved better overall levels of background match than crabs in any of the other three treatments. Meanwhile crabs exposed to ship noise pollution, regardless of temperature, exhibited lower rates of luminance change and poorer overall background matching at the end of the 6-wk experimental period than those experiencing ambient sounds played at the same intensity under warm temperatures. Our results represent a potential antagonistic interaction between the 2 stressors, with anthropogenic ship noise overriding impacts of temperature when applied in combination in this case, although it is difficult to draw conclusions about the strength of this interaction without further testing of treatments at different magnitudes.

The significant reduction in rate and efficacy of background match in ship noise treatment groups seen here may result in severe fitness consequences for juvenile shore crabs. Increased predation rates and reduced survival have been recorded in individuals poorly matched to their backgrounds, including snowshoe hares, isopods, and chameleon prawns, and it has been suggested that such effects could lead to population level declines without rapid adaptation (Hultgren and Mittlestaedt 2015; Zimova et al. 2016, Mynott 2019). Camouflage mismatch may be perceived differently by predators with differing visual systems. Here, we use an avian visual model to analyze luminance change perceptible to a common crab predator. Further studies could incorporate a wider range of visual systems to gain an in depth understanding of how such mismatches may impact species in the wild.

In accordance with findings from previous studies (Wale et al. 2013a; Carter et al. 2020) those crabs experiencing ship noise playback were less likely to retreat from a simulated predatory attack than those exposed to ambient playback at the same amplitude. However, retreat times of crabs that did respond were slowest in Cold-Ambient treatment groups, suggesting that higher temperatures have the potential to decrease response latency.

Although adult and larval thermal tolerance of *C. maenas* has been recorded (Kelley et al. 2013), studies specifically focused on the thermal tolerance ranges of settling juveniles have not been performed. However, previous work has suggested that 15 °C to 20 °C is the optimum range for background matching in this life stage (Mynott 2019). In past studies, crabs matched their substrate best at 20 °C on both dark and light substrates but color change on white broke down at 25 °C, potentially due to thermal stress (Mynott 2019). Powell (1962) also observed impaired match of crabs held on dark substrates at 30 °C. Here, we did not find a break down in color change at 24 °C under ambient noise, but crabs changed more slowly when in the presence of ship noise. Noise pollution has been associated with stress in crustaceans (e.g. Wale et al. 2013a). Given that chromatophore function is controlled endocrinologically, changes to the endocrine response in crabs exposed to ship noise treatments, and resultant alterations to hormone regulation, could be responsible for the lower rates and efficacy of background match in crabs in these treatments (Fingerman 1970; Duarte et al. 2017; Carter et al. 2020). The magnitude of this response to noise pollution appears to be sufficient to negate the improvement in camouflage brought about by warm temperatures alone. This occurs at temperatures that are just above the current upper limit of summer open water temperatures locally (19 to 21 °C) but within the range reached in isolated rock pools at low tide.

As a species with a wide thermal tolerance range, it is possible that *C. maenas* will be less sensitive to thermal stress than species of crab with narrower limits, or poorer ability to acclimate to changing temperatures (Stillman 2003). However, wide thermal ranges are relatively common across intertidal species of crab due to regularly fluctuating temperatures in rocky shore environments (Stillman and Somero 1998, Stillman 2002). It may be possible therefore to extrapolate the results here to other species with similar known thermal limits. Although auditory perception and detection mechanisms are less well understood among crustaceans (Popper et al. 2001), and frequency ranges of specific species vary, crustaceans are generally most sensitive to low frequencies (Radford et al. 2022). It may therefore be expected that boat noise falls within auditory detection thresholds of many crustacean species, although exact responses may be hard to predict due to differing sensitivities between species.

Crab growth rate, overall weight, and carapace width were highest under raised temperature treatments by the end of the 6-wk experimental period, with no impact of noise treatment. The lack of impact of noise on growth rates could indicate that the mechanisms negatively impacted by ship noise when considering chromatophore function are not vital for organism growth. This may point toward an endocrinological impact of noise pollution, as opposed to simply the alteration of metabolic rate (Fuhrmann et al. 2011). Alternatively, prioritization of resource allocation to growth over camouflage may have occurred, demonstrating a potential plasticity in life-history traits under stress. This response has been widely recorded in organisms exposed to stress, for example, the fall armyworm (*Spodoptera frugiperda*) may alter allocation energy to reproduction or growth under stressful temperature conditions, showing a plasticity of life history strategy (Wu et al. 2022). This ability could have several advantages for juvenile organisms across taxa, not least in shortening generation times, intraspecific competition, foraging ability, and development of more robust morphological antipredator defenses. Future studies exploring the potential for this rapid plasticity would be valuable, particularly in light of the shore crab’s global impact as an invasive species (Ens et al. 2022). Comparisons of shore crab populations in different habitat types (estuarine, rocky shore, subtidal) with regards to their response to stress would provide more detailed insight into their resilience in the face of habitat change. This would also be useful given that crabs from different habitats can vary in coloration, camouflage type, and variability (Stevens et al. 2014; Price et al. 2019), and such individuals may respond differently to stressors.

Maladaptive responses to stressor exposure are particularly concerning when they affect behaviors key to survival. The reduced ability of crabs to respond appropriately to predators in the presence of ship noise could result in mortality and has now been demonstrated across multiple studies (Wale et al. 2013a; Radford et al. 2022) and in a range of other species (Siemers and Schaub 2010; Bruintjes and Radford 2013; Simpson et al. 2015, 2016a,b). It has been posited that the physical qualities of ship noise in comparison with natural sounds distract attention from antipredator vigilance behaviors, which reduces capacity for predator detection (Chan et al. 2010; Wale et al. 2013a, b; Carter et al. 2020), and they could also result in the favoring of vital noise avoidance behaviors to preserve sensory systems. Of those crabs that did respond, Cold-Ambient treatment crabs retreated significantly slower than any other treatment group. It is possible that crabs that did detect and respond to the predator were more stressed by ship noise, and so responded faster than those crabs experiencing only ambient noise. Furthermore, lower temperatures are commonly associated with slower movement and reduced metabolic rates in crustaceans (Weinstein 1998), and as such this could have served to increase response latency. Unpredictable consequences of stressor combinations like this demonstrate the need for future studies to further explore cross-modal impacts of multiple stressors.

Increased stress resultant from noise pollution may incur a metabolic cost, and therefore available energy for flight response may be reduced (Wale et al. 2013a; Simpson et al. 2015; Ruiz-Ruiz et al. 2020). Higher metabolic rates in crabs experiencing raised temperatures may prompt increased energy demands and foraging necessity, meaning that the trade-off between remaining in the open and the possibility of being attacked is more heavily weighted in favor of foraging opportunity (Wallace 1972). Resource allocation in the trade-off between foraging and predation risk has been well studied, and stress may alter this relationship (McNamara and Buchanan 2005). For example, plasticity in this allocation has been demonstrated in marine intertidal snails in response to changes in temperature (Miller et al. 2014). This motivation is, however, difficult to extrapolate from our study. Although some crabs were observed to be sifting the substrate in search of food, no food was provided, and is unclear whether crabs given the opportunity to forage successfully within the trial period would have prioritized this over predation likelihood or not. Mud crabs demonstrate reduced foraging activity during playback of predator sounds and respond more strongly to auditory predator cues than to chemical predator cues (Hughes et al. 2014). If this dominant role of auditory predator detection is shared by shore crabs then masking of these cues by ship noise could be even more detrimental to crab survival.

Behavioral plasticity in response to stress has been demonstrated in many single stressor studies. For example, whales alter their call amplitude in response to masking by ship noise (Parks et al. 2007, 2011), while long-term exposure to adverse conditions may prompt irreversible shifts in species range. This is a behavior particularly evident in the face of climate change, with global distributions of marine species shifting by ever larger increments (Mieszkowska et al. 2006; Sorte et al. 2010; Wallingford et al. 2020). Stressor interaction may complicate these responses and make behaviors more difficult to predict, and to manage. In scenarios where multiple stressors have an antagonistic effect, the removal of one stressor may in fact worsen the overall impacts of environmental change on an ecosystem (Côté and Darling 2010). For example, sedimentation is known to reduce coral survival, however, in conditions of extreme UV exposure, increased sedimentation may block harmful impacts of UV and prevent coral bleaching. Therefore, management of sedimentation may result in a net negative impact on coral reefs compared with no management (Anthony et al. 2007; Brown et al. 2013). It is also possible that changes in temperature may directly link to other changes in the visual environment itself. For example, higher temperatures may impact seaweed growth and survival (Harley et al. 2012), changing the visual environment and hence camouflage efficacy.

Contemporary studies are increasingly examining the effects of multiple stressor interactions on study species, with the realization that species in natural environments are subject to stressors that vary in source, as well as spatially and temporally. This study is one of the few of its kind to combine both a local and a global stressor to test their interaction. Future stressor interaction studies should continue to combine local and global stressors and seek to study their potential impact on behaviors not directly related to the stressors in question. In an ever-changing world, where anthropogenic drivers of environmental change are fluctuating and complex, ecologically relevant study of stressor interaction and cross-modal impacts is crucial.

## Supplementary Material

arae085_suppl_Supplementary_Materials

## Data Availability

Analyses reported in this article can be reproduced using the data provided by Coles et al. (2024).
