# Supplementary material for: Interaction between anthropogenic stressors affects antipredator defense in an intertidal crustacean
Source: Behav Ecol. 2024 Oct 10;35(6):arae085. doi: 10.1093/beheco/arae085 (PMC11520746; doi:10.1093/beheco/arae085)
Supplement: arae085_suppl_Supplementary_Materials [file arae085_suppl_supplementary_materials.doc]

**Supplementary Online Materials**

**Interaction between anthropogenic stressors affects antipredator defence in an intertidal crustacean**

**S1. Sound treatments**

Sound recordings were provided by Wale, Simpson and Radford from their studies (2013 a,b). Originally taken at the UK ports of Plymouth, Portsmouth and Gravesend, recordings were made at each location to capture ambient background noise, as well as the sound of a ship passing at a distance of approximately 200 m (of an LPG tanker, container ship and ferry respectively). For full details of recording and sound modification procedures, see Wale et al. [8,9]. Further sound treatment modifications were made using Audacity ® (Audacity Team, 2020), in accordance with a study by Carter et al. (2020), so that all played at a comparable amplitude. Individual tracks were looped to create six 5-minute playbacks, each with a 30 second fade in and out.

Table S1: Mean sound levels of each combined treatment recorded 60 mm above tank base, averaged across the centre, far left and far right of the tank. RMS(A): Root Mean Squared Average, A-weighted (A-weighting is commonly used in environmental pollution studies to standardise sound with respect to human hearing response); dbFS: decibels relative to full scale.

| *Noise* | *RMS (A)* | *RMS dBFS* | *Peak dBFS* |
| --- | --- | --- | --- |
| *Ambient* | -40.65 | -41.13 | -19.29 |
| *Ship* | -37.96 | -40.8 | -21.3 |


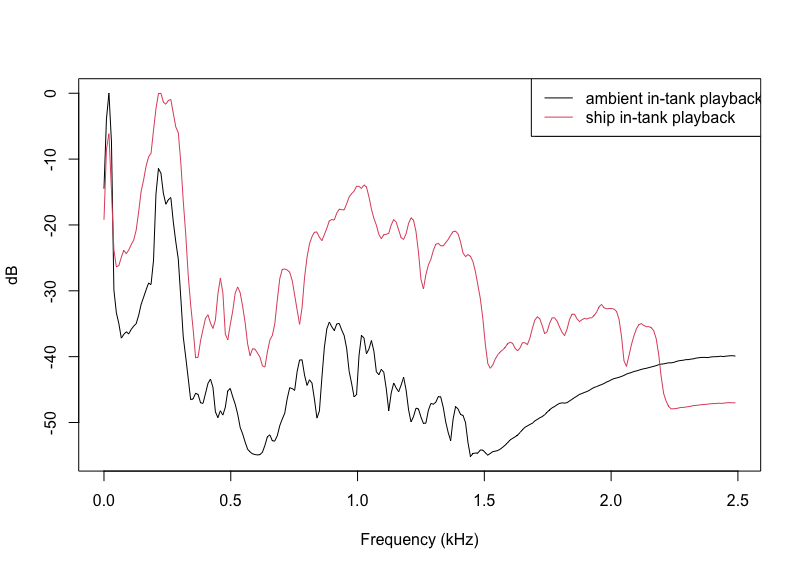

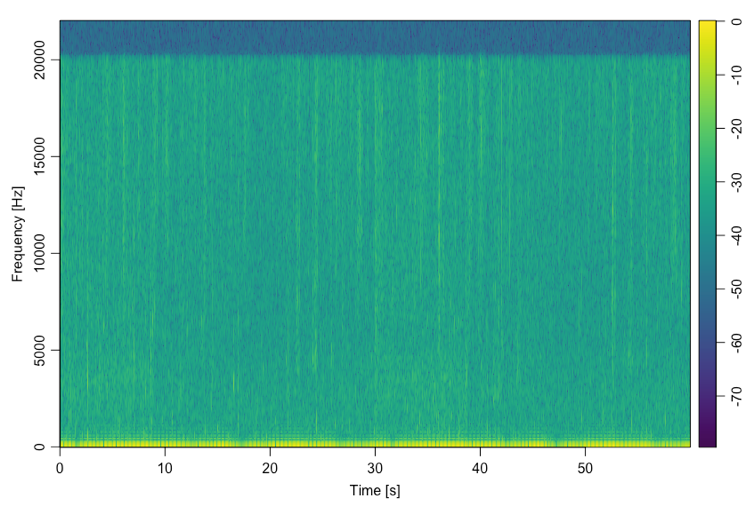

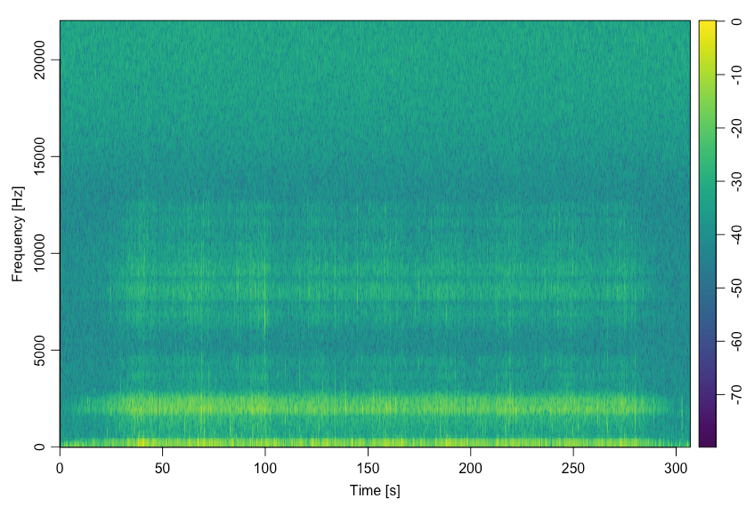

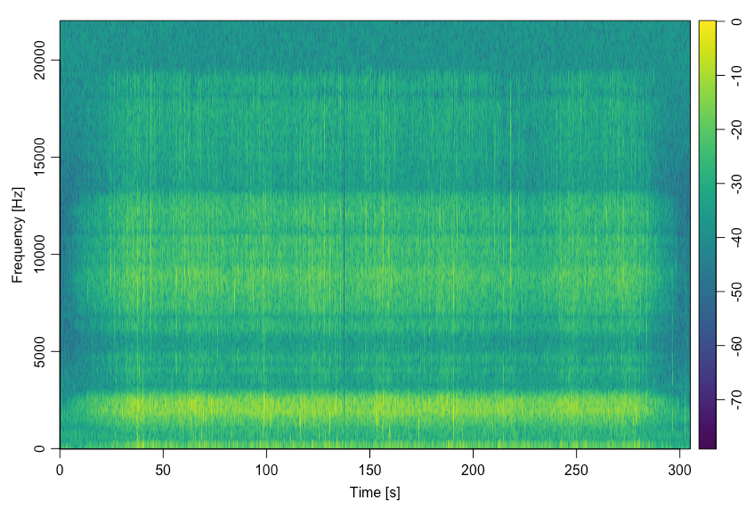

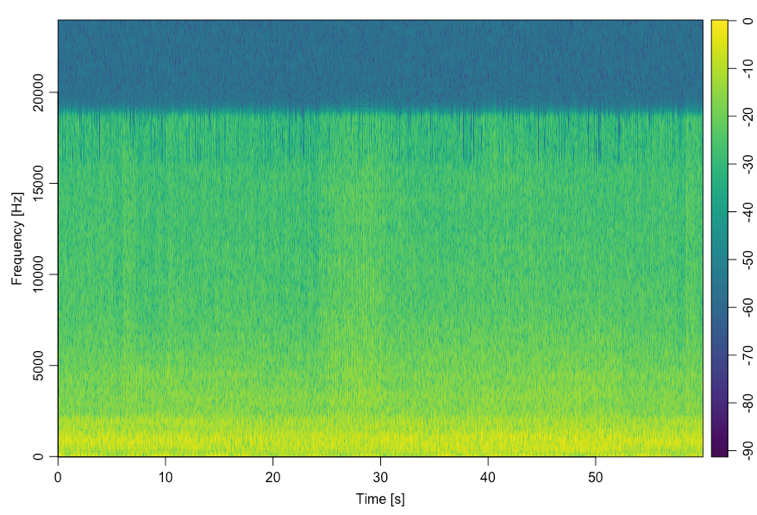


**Figure S1**: Sound spectra for original recordings and in-tank playback of sound treatments. **a)** Sound profile spectrograph for recorded ship and ambient in-tank playback. Ship noise denoted in red, ambient in black. FFT size:1024, window size cropped to 2.5kHz to reflect known crab auditory detection thresholds. **b, c, d and e)** spectrograms of original recordings of ship **(b)** and ambient **(c)** sounds, and in tank playback of ship **(d)** and ambient **(e)**sounds.

**a)**

**b)**

**c)**

**d)**

**e)**

**S2. Initial luminance**

Carapace colouration and size of crabs upon collection was naturally variable, and there was a small but significant difference in the starting luminance of crabs, which was first recorded following the one-week acclimation period, with a higher mean luminance of Hot treatment crabs (Kruskal-Wallis; X^2^_1_ = 6.28, p=0.01). This may have been influenced by the acclimation week, where the gradual increase of temperatures in Hot treatment tanks over the 7-day period could have prompted disproportionate premature lightening of crab carapaces, compared with those in cold temperature treatments.

**S3: Full statistical results:**

**Table S2: Model comparisons using the chi squared analysis of variance for background match of crabs to substrate:**

| **Focal Term** | **df** | **Chi-squared statistic. (x2)** | **p-value** |
| --- | --- | --- | --- |
| **Noise*Temperature** | 432 | -0.0042534 | 0.02997 |
| **Temperature** | 433 | 0.0054957 | 0.01419 * |
| **Noise** | 432 | 0.0022241 | 0.118 |
| **Week** | 432 | -0.097099 | < 2.2e-16 *** |
| **Moult (y/n)** | 432 | -0.0095909 | 0.001117 ** |
| **Number of moults** | 432 | -0.020764 | 1.621e-06 *** |
| **Carapace width** | 429 | 0.00068577 | 0.3835 |
| **Carapace weight** | 430 | 0.00093685 | 0.3083 |

**Table S3:** Pairwise comparisons for background match between treatment groups after 6 weeks, using lsmeans package.

| **Contrast** | **Estimate** | **SE** | **df** | **t-ratio** | **p-value** |
| --- | --- | --- | --- | --- | --- |
| **Cold Ambient - Hot Ambient** | 0.013 | 0.004 | 431 | 3.228 | 0.007 |
| **Cold Ambient - Cold Ship** | 0.001 | 0.004 | 431 | 0.334 | 0.987 |
| **Cold Ambient - Hot Ship** | 0.002 | 0.004 | 431 | 0.471 | 0.965 |
| **Hot Ambient - Cold Ship** | -0.011 | 0.004 | 431 | -2.983 | 0.016 |
| **Hot Ambient - Hot Ship** | -0.01 | 0.004 | 431 | -2.657 | 0.041 |
| **Cold Ship - Hot Ship** | 0.001 | 0.004 | 431 | 0.16 | 0.999 |

**Table S4:** Model comparisons using the chi squared analysis of variance for antipredator retreat times:

| **Focal Term** | **df** | **Chi-squared statistic. (x2)** | **p-value** |
| --- | --- | --- | --- |
| **Previous experience** | 118 | 2.181 | 0.153 |
| **Noise*Temperature** | 119 | 10.34 | 0.002 |
| **Temperature** | 120 | 2.478 | 0.138 |
| **Noise** | 120 | 1.186 | 0.305 |
| **Noise*Temperature*Previous exposure** | 115 | 4.655 | 0.398 |

**Table S5:** Pairwise comparisons for antipredator retreat times between treatment groups, using lsmeans package.

| **Contrast** | **Estimate** | **SE** | **df** | **t-ratio** | **p-value** |
| --- | --- | --- | --- | --- | --- |
| **Cold Ambient – Hot Ambient** | -0.052 | 0.019 | 119 | -2.728 | 0.036 |
| **Cold Ambient – Cold Ship** | -0.043 | 0.018 | 119 | -2.395 | 0.084 |
| **Cold Ambient – Hot Ship** | -0.013 | 0.014 | 119 | -0.9 | 0.805 |
| **Hot Ambient – Cold Ship** | 0.008 | 0.024 | 119 | 0.363 | 0.984 |
| **Hot Ambient – Hot Ship** | 0.039 | 0.021 | 119 | 1.829 | 0.265 |
| **Cold Ship – Hot Ship** | 0.030 | 0.02 | 119 | 1.481 | 0.452 |

**Table S6:** Model comparisons using the chi squared analysis of variance for antipredator retreat response (Y/N):

| **Focal Term** | **df** | **Chi-squared statistic. (x2)** | **p-value** |
| --- | --- | --- | --- |
| **Previous experience** | 289 | 0.448 | 0.504 |
| **Noise*Temperature** | 290 | 0.081 | 0.776 |
| **Temperature** | 291 | 2.061 | 0.151 |
| **Noise** | 291 | 4.449 | 0.035 |
| **Noise*Temperature*Previous exposure** | 290 | -1.11 | 0.893 |

**Table S7:** Pairwise comparisons for antipredator retreat response (Y/N) between treatment groups, using lsmeans package.

| **Contrast** | **Estimate** | **SE** | **df** | **t-ratio** | **p-value** |
| --- | --- | --- | --- | --- | --- |
| **Cold Ambient - Hot Ambient** | 0.277 | 0.332 | Inf | 0.835 | 0.838 |
| **Cold Ambient - Cold Ship** | 0.437 | 0.332 | Inf | 1.317 | 0.552 |
| **Cold Ambient - Hot Ship** | 0.850 | 0.342 | Inf | 2.484 | 0.062 |
| **Hot Ambient - Cold Ship** | 0.160 | 0.335 | Inf | 0.477 | 0.964 |
| **Hot Ambient - Hot Ship** | 0.573 | 0.345 | Inf | 1.659 | 0.346 |
| **Cold Ship - Hot Ship** | 0.413 | 0.345 | Inf | 1.197 | 0.628 |

**S9. Supplemental references**

Wale, M.A., Simpson, S.D., Radford, A.N., 2013. Noise negatively affects foraging and antipredator behaviour in shore crabs. Animal Behaviour 86, 111–118. <https://doi.org/10.1016/j.anbehav.2013.05.001>

Wale, M.A., Simpson, S.D., Radford, A.N., 2013. Size-dependent physiological responses of shore crabs to single and repeated playback of ship noise. Biology Letters 9, 20121194. <https://doi.org/10.1098/rsbl.2012.1194>
